# How to prevent the detrimental effects of two months of bed-rest on muscle, bone and cardiovascular system: an RCT

**DOI:** 10.1038/s41598-017-13659-8

**Published:** 2017-10-13

**Authors:** Andreas Kramer, Albert Gollhofer, Gabriele Armbrecht, Dieter Felsenberg, Markus Gruber

**Affiliations:** 1 Sensorimotor Performance Lab, University of Konstanz, 78457 Konstanz, Germany; 2 Department of Sports and Sports Science, University of Freiburg, Schwarzwaldstr, 175, 79117 Freiburg, Germany; 3 Centre of Muscle and Bone Research, Charité University Medicine Berlin, Hindenburgdamm 30, 12200 Berlin, Germany

## Abstract

Physical inactivity leads to a deconditioning of the skeletal, neuromuscular and cardiovascular system. It can lead to impaired quality of life, loss of autonomy, falls and fractures. Regular exercise would be a logical remedy, but the generally recommended high-volume endurance and strength training programs require a lot of time and equipment. In this randomized controlled study with 23 healthy participants, we established that a short, intensive jump training program can prevent the large musculoskeletal and cardiovascular deconditioning effects caused by two months of physical inactivity during bed rest, particularly the loss of bone mineral mass and density, lean muscle mass, maximal leg strength and peak oxygen uptake. The jump training group showed no significant changes with respect to these indicators of musculoskeletal and cardiovascular health after 60 days of bed rest, whereas the control group exhibited substantial losses: up to −2.6% in tibial bone mineral content and density, −5% in leg lean mass, −40% in maximal knee extension torque and −29% in peak oxygen uptake. Consequently, we recommend jump training as a very time-efficient and effective type of exercise for astronauts on long-term space missions, the elderly and sedentary populations in general.

## Introduction

Physical inactivity can have strong deconditioning effects on the human body, in particular on bones, muscles and the cardiovascular system^1,2^, in severe cases even leading to osteoporosis^3^, sarcopenia^4^ and cardiovascular diseases^5^. It has also been established that it can lead to decreased mobility, reduced capacity for activities of daily living, impaired quality of living, falls and fractures^6^. This deconditioning due to a lack of adequate loading can be observed in different scenarios: it is a problem for astronauts whose bodies are no longer subjected to gravitational loading^7^, it is also true for elderly, especially if ill and bed-ridden^8^, and becomes an increasing problem in modern societies with a predominantly sedentary lifestyle^2^. The lower extremities seem to be predominantly affected, which is consistent with the fact that the lower extremities bear most of the body weight and will therefore be most affected by reduced weight-bearing activity^9^.

To counteract this deconditioning problem, regular physical exercise is a logical remedy, and its efficacy has been demonstrated time and again^10^. Of course, not every type of exercise is suitable. The most common recommendation against cardiovascular deconditioning (concerning primarily maximal oxygen uptake capacity, heart rate, blood pressure, and atherogenic risk factors) is moderate-intensity aerobic exercise for at least 3 times per week with a total duration of several hours; against losses in muscle mass, strength and power, resistance training for at least 2 times per week; to prevent osteoporosis, high-impact exercise such as running or jumping^10^. It is evident that it would require considerable time and a multitude of training devices to follow all these recommendations. This might be one of the reasons that despite overwhelming evidence for the beneficial effects of exercise, adherence in the general population to the recommendations outlined above is very low^11^.

Consequently, one possible remedy would be a training program that counteracts many of the deconditioning effects at once and does not require much time. One type of exercise that has the potential to fulfil these criteria is jumping: jumping is a high-intensity, low-volume type of training that does not require much time, yet, reactive jumping induces high strain and strain rates^12^, which have been suggested to be key determinants for bone strength^13,14^. In addition, jump training has repeatedly been shown to increase leg muscle strength^15^. If used as a form of high intensity interval training, jumps can also be used to counteract cardiovascular deconditioning, as recent studies have shown that high-intensity interval training has similar beneficial effects on maximum oxygen uptake capacity and cardiovascular health as traditional high-volume, moderate-intensity aerobic training^16^.

Therefore, the aim of the present study was to assess the efficacy of a short high-intensity jump training as a countermeasure for the deconditioning effects of physical inactivity. We hypothesized that there would be significant differences between training and control group after bed rest with respect to bone mineral content and density of the tibia, leg lean mass, maximal strength of the leg extensors, and peak oxygen uptake capacity.

## Results

**Figure 1:**
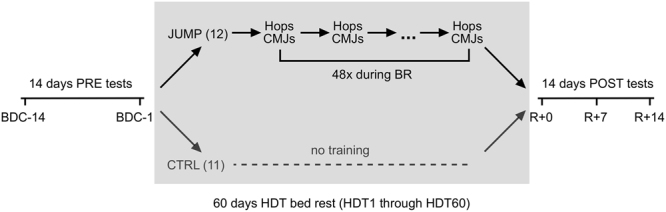
Study overview. Prior to the bed rest phase, participants spent 14 days in the bed rest facility for familiarisation and baseline data collection (BDC-14 through BDC-1). In the morning of the first head-down tilt bed rest day (HDT1), participants were randomly assigned to either the training group (JUMP, total of 48 training sessions during the 60 days of bed rest) or the control group (CTRL). After the 60 days of HDT bed rest, participants were re-ambulated and stayed for an additional 15 days in the bed rest facility for measurements and recovery (R + 0 through R + 14).

**Figure 2:**
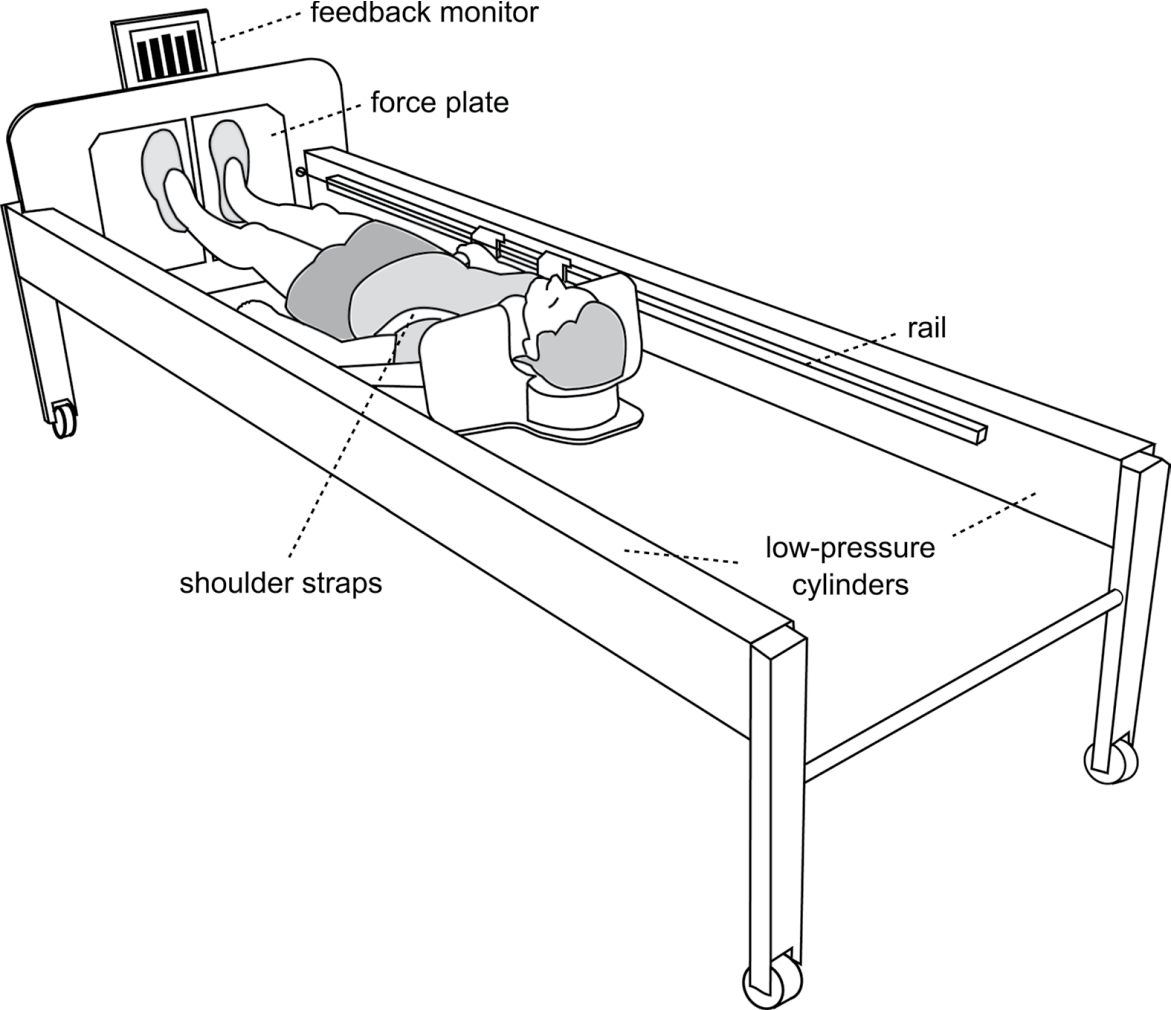
The training device (sledge jump system, SJS). The participant is fixed to the wooden sledge with shoulder straps, and his thighs rest on additional straps. The straps are attached to the rails and can slide in the direction of the rails with minimal friction. The participant is pulled towards the force plates by the force generated by the low-pressure cylinders.

For this randomized controlled study, 23 male participants were subjected to 60 days of strict bed rest. In addition, two weeks before and after the bed rest phase, participants were confined to the bed rest facility for familiarization, measurements and recovery, see Fig. 1. Subjects were randomly allocated to the jump training group (JUMP, 12 participants) or the control group (CTRL, 11 participants). Participants in the training group trained 5-6x per week (see Fig. 2), and on average, each training session consisted of 6 × 12 jumps, with an exercise duration of about three minutes.

**Figure 3:**
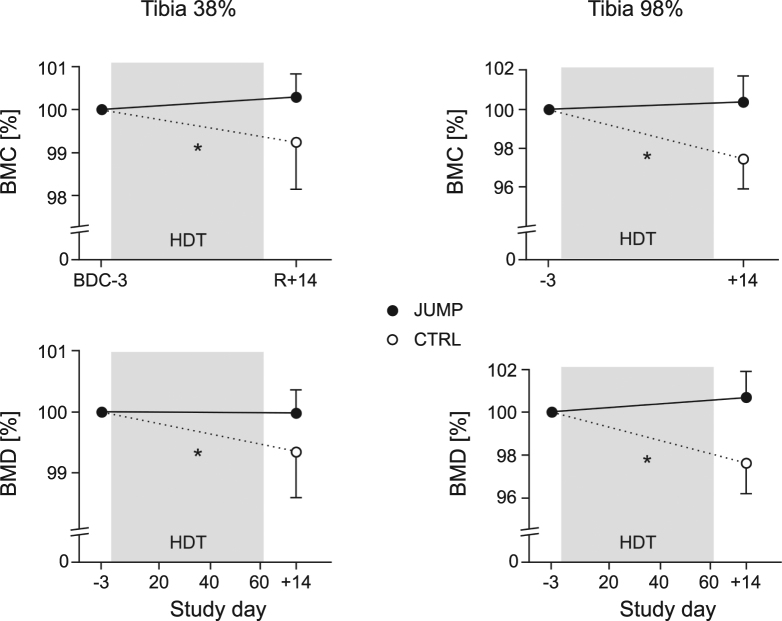
Changes in total bone mineral content (BMC) and total bone mineral density (BMD) with respect to baseline (BDC-3) at two sites (at 38% and 98% of tibia length). Full circles represent the mean and standard deviation of the training group (JUMP, N = 12), open circles the control group (N = 11). A *symbol denotes a significant group*time interaction effect.

Changes in bone mass and density of the tibia were assessed via four cross-sectional images at 4%, 38%, 66% and 98% of the tibia length using peripheral quantitative computed tomography scans (pQCT). Similarly, cross-sectional images of the radius were obtained at 60% of radius length. When comparing the tibia pQCT images from before bed rest to the ones taken two weeks after the end of bed rest, there were no significant changes in bone mineral content or density for the training group, but a decrease in the control group, with significant group*time effects and large effect sizes for all sites and parameters, see Table 1 and Fig. 3. As expected, there were no changes in the radius, which served as an upper body control (no group*time effects, no main effects of time, p = 0.28 for bone mineral content (BMC) and p = 0.83 for bone mineral density (BMD)).

**Table 1:** Bone and muscle mass.

Tibia	JUMP pre	JUMP post	CTRL pre	CTRL post	Interaction group*time	Hedge’s g
BMC at 4% [mg/mm]	423 ± 55	422 ± 54 n.s. + 0.0 ± 1.6%	431 ± 49	421 ± 52 −2.5 ± 1.6%	F_1,21_ = 14.7 p < 0.001	1.50
BMD at 4% [mg/cm^3^]	322 ± 34	320 ± 34 n.s. −0.5 ± 0.4%	326 ± 35	319 ± 37 −2.2 ± 1.9%	F_1,21_ = 5.3 p = 0.03	0.94
BMC at 38% [mg/mm]	426 ± 55	427 ± 55 n.s.+ 0.3 ± 0.6%	423 ± 36	420 ± 36 −0.8 ± 1.1%	F_1,21_ = 7.8 p = 0.01	1.18
BMD at 38% [mg/cm^3^]	896 ± 47	896 ± 45 n.s.+ 0.0 ± 0.4%	903 ± 58	897 ± 60 −0.6 ± 0.7%	F_1,21_ = 5.8 p = 0.03	1.01
BMC at 66% [mg/mm]	466 ± 53	466 ± 52 n.s.+ 0.2 ± 0.3%	469 ± 49	465 ± 49 −0.7 ± 0.6%	F_1,21_ = 19.6 p < 0.001	1.97
BMD at 66% [mg/cm^3^]	738 ± 50	739 ± 49 n.s.+ 0.1 ± 0.5%	716 ± 57	710 ± 55 −0.8 ± 0.6%	F_1,21_ = 12.6 p = 0.002	1.67
BMC at 98% [mg/mm]	829 ± 87	832 ± 86 n.s.+ 0.3 ± 1.5%	880 ± 123	858 ± 125 −2.6 ± 1.5%	F_1,21_ = 22.7 p < 0.001	1.90
BMD at 98% [mg/cm^3^]	213 ± 21	214 ± 20 n.s.+ 0.7 ± 1.3%	227 ± 32	221 ± 32 −2.4 ± 1.5%	F_1,21_ = 27.9 p < 0.001	2.25
Radius BMC at 60% [mg/mm]	123 ± 14	123 ± 15 n.s. −0.1 ± 1.3%	131 ± 16	131 ± 16 −0.4 ± 0.9%	F_1,21_ = 0.4 p = 0.52	0.28
Radius BMD at 60% [mg/cm^3^]	847 ± 74	846 ± 78 n.s. −0.1 ± 0.9%	828 ± 65	830 ± 63 + 0.2 ± 1.1%	F_1,21_ = 0.3 p = 0.58	−0.30
DXA leg lean mass [kg]	19.4 ± 1.4	19.3 ± 1.5+ 0 ± 3% n.s.	19.6 ± 2.4	18.6 ± 2.2 −5 ± 3%	F_1,21_ = 14.0 p = 0.001	1.58

Results of the pQCT and DXA measurements, separately for the training group (JUMP) and the control group (CTRL), once during baseline (BDC-3 for the tibia, BDC-13 for the radius) and once during recovery (R + 14 for pQCT, R + 7 for DXA). The percent values reflect the changes observed at R + 14 compared to baseline. Total bone mineral content (BMC) and total bone mineral density (BMD) were assessed at four tibia sites, at 4%, 38%, 66% and 98% of tibia length with respect to the tibia’s distal end. For the training group, none of the R + 14-values were statistically significantly different from baseline (n.s.). Hedge’s g refers to the mean changes in the training group versus the mean changes in the control group.

**Figure 4:**
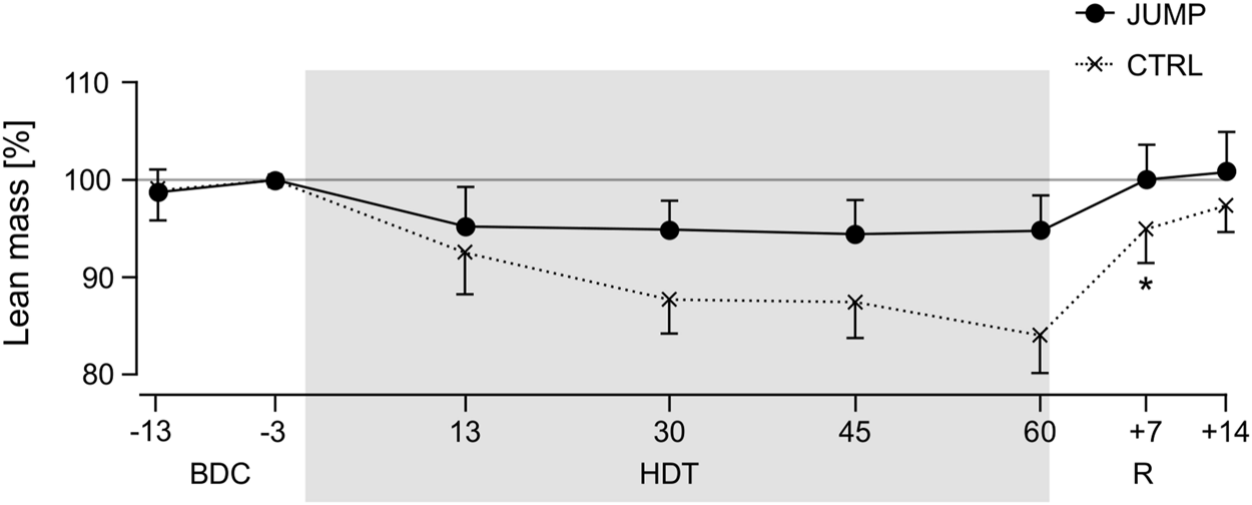
Leg lean mass based on DXA analyses, normalized to BDC-3 values. Solid lines represent mean (and standard deviation) for the training group (JUMP, N = 12), broken lines for the control group (CTRL, N = 11). Two measurements were taken during baseline data collection (BDC-13 and BDC-3), four during bed rest (HDT13, HDT30, HDT45, HDT60) and two during recovery (R + 7, R + 14). A *symbol denotes a significant group*time interaction effect.

Leg muscle mass was determined with dual energy x-ray absorptiometry (DXA) scans performed twice during the two weeks of baseline data collection before bed rest (BDC-13 and BDC-3), four times during head-down tilt bed rest (HDT13, HDT30, HDT45 and HDT60), and twice during the two weeks of recovery after bed rest (R + 7 and R + 14). The DXA scans revealed a decrease in lean leg mass for the control group, but again no changes for the training group (p = 0.79; significant group*time effect, p = 0.001; see Fig. 4 and Table 1).

**Figure 5:**
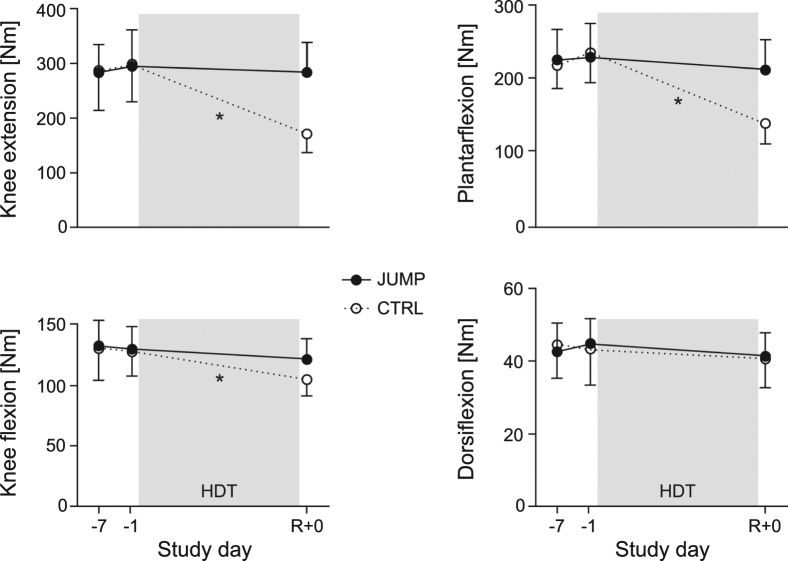
Results of the isometric MVC recordings, full circles representing mean and standard deviation of the training group (JUMP, N = 12), open circles representing the control group (CTRL, N = 11). First data point (BDC-7) represents the familiarization session, the second one represents the measurement directly before bed rest (BDC-1), and the last one the measurement directly after bed rest (R + 0). A *symbol denotes a significant group*time interaction effect.

As muscle function can significantly decrease with no changes in total muscle mass due to neural adaptations or changes in muscle fibre properties, we also assessed maximal muscle strength and local muscle endurance directly before bed rest (BDC-1) and directly after bed rest (R + 0). Isometric maximal voluntary contraction (MVC) torque was recorded during knee extension, knee flexion, plantar flexion and dorsiflexion. In addition to the MVC tests for the lower extremity, maximal handgrip strength was recorded as a control measure of upper extremity strength. We found that maximal leg strength considerably decreased in the control group, but could be maintained by the training during knee extension and knee flexion (no significant pre-post differences for JUMP, p = 0.26 and p = 0.14, respectively), and was attenuated during plantar flexion, see Table 2 and Fig. 5. While MVC tests reflect the muscles’ ability to generate high forces for a short amount of time, sustained muscle contractions reflect the muscles’ ability to generate force over a period of time, i.e., local muscle endurance. This was tested during sustained submaximal isometric contractions of the knee extensors. The analyses revealed that knee extensor torque over 90 s did not change significantly in the training group due to bed rest (p = 0.42), but was significantly lower in CTRL, with a significant group*time interaction effect, see Table 2. As expected, handgrip strength tests showed no significant changes in either group (see Table 2; no group*time interaction, p = 0.52, no time effect, p = 0.31).

**Table 2:** Strength and aerobic capacity.

MVC	JUMP BDC-1	JUMP R + 0	CTRL BDC-1	CTRL R + 0	Interaction group*time	Hedge’s g
Knee extension [Nm]	294 ± 67	284 ± 55 −3 ± 9% n.s.	298 ± 69	171 ± 34 −41 ± 11%	F_1,21_ = 38.5 p < 0.001	3.86
Knee flexion [Nm]	129 ± 19	121 ± 18 −6 ± 14% n.s.	126 ± 18	105 ± 14 −16 ± 13%	F_1,21_ = 3.0 p = 0.10	0.77
Plantar flexion [Nm]	228 ± 47	210 ± 42 −8 ± 10%	235 ± 41	138 ± 29 −40 ± 12%	F_1,21_ = 33.2 p < 0.001	3.01
Dorsiflexion [Nm]	45 ± 7	41 ± 7 −8 ± 8%	43 ± 10	40 ± 8 −6 ± 9%	F_1,21_ = 0.07 p = 0.79	−0.17
Knee extension torque over 90 s [Nm*s]	10831 ± 2484	10436 ± 2083 −3 ± 12% n.s.	11215 ± 2703	6353 ± 1491 −42 ± 9%	F_1,19_ = 33.7 p < 0.001	3.76
Handgrip strength [kg]	51 ± 8	53 ± 7 + 5 ± 14% n.s.	51 ± 5	52 ± 5 + 1 ± 7%	F_1,21_ = 0.4 p = 0.52	0.33
**Spiroergometry**	**JUMP BDC-8**	**JUMP R + 1**	**CTRL BDC-8**	**CTRL R + 1**	Interaction group*time	**Hedge’s g**
rel. VO2peak [ml/(min*kg)]	42.7 ± 8.7	39.8 ± 6.6 −5 ± 15% n.s.	50.1 ± 7.3	35.3 ± 7.1 −29 ± 11%	F_1,17_ = 19.2 p < 0.001	1.84
abs. VO2peak [l/min]	3.32 ± 0.76	2.99 ± 0.53 −8 ± 14% n.s.	3.84 ± 0.68	2.57 ± 0.48 −32 ± 11%	F_1,17_ = 15.8 p < 0.001	1.88
Peak power [W]	280 ± 40	245 ± 40 −12 ± 8%	325 ± 41	228 ± 28 −29 ± 10%	F_1,17_ = 16.3 p < 0.001	1.80
Peak heart rate [bpm]	185 ± 7	187 ± 6+2 ± 5% n.s.	188 ± 7	188 ± 10 0 ± 4%	F_1,16_ = 0.65 p = 0.43	0.4

Results of the MVC and spiroergometry tests, separately for the training group (JUMP) and the control group (CTRL), once during baseline (BDC-1 or BDC-8) and once directly after bed rest (R + 0 or R + 1). The percent values reflect the changes observed after bed rest compared to baseline. For the training group, n.s. denotes the R + 0-values that were not statistically significantly different from baseline.

The gross performance of the cardiovascular system was assessed with a cycle ergometer step test, once before bed rest (BDC-8) and once after bed rest (R + 1). The load was increased every minute in steps of 25 W until volitional exhaustion occurred. Oxygen uptake and carbon dioxide emission as well as heart rate were recorded, and peak oxygen uptake was determined as a measure of aerobic capacity. In the training group, peak oxygen uptake capacity (VO2peak) was maintained (see Table 2, no significant pre-post difference for the training group, p = 0.16 for relative VO2peak, p = 0.051 for absolute VO2peak), and the reduction in peak power was attenuated (significant group*time interaction, p < 0.001), whereas the control group showed a pronounced decrease. Maximal heart rate during the ergometer test did not change for either group (no group*time interaction effect, p = 0.23, no time effect, p = 0.49).

## Discussion

The short but intensive jump training program was successful in preserving bone mass, maximal muscle force and peak oxygen uptake capacity throughout the 60 days of strict physical inactivity during bed rest.

It is remarkable that bone mineral content and density of the tibia could be maintained at all measured sites by the jump training during bed rest, while the various countermeasures used in previous bed rest studies were sometimes not even able to attenuate bone loss. Non-exercise countermeasures such as nutrition or pharmacological interventions were in general least successful in maintaining bone mass. For example, increasing protein intake by 60% during 60 days of bed rest yielded no significant benefit for BMC compared to the control group in the Women’s International Space Simulation for Exploration study (WISE)^17^. Similarly, doubling calcium intake did not have a significant effect on bone turnover markers during short-term bed rest^18^. Bisphosphonate treatment (pamidronate) during 90 days of bed rest in the long-term bed rest study (LTBR) could only attenuate the reduction in BMC by about 50%^19^. Results from bed rest studies using some form of exercise as a countermeasure were in general more promising, but most training interventions were still hardly able to maintain bone mass: the combination of aerobic exercise (treadmill) and resistance exercise (flywheel) used in the WISE study showed no significant effect compared to control^17^. The maximal concentric and eccentric resistance training with the flywheel used in the LTBR study had a significant effect, but could only attenuate the BMC reduction by roughly 50%^19^. The whole body vibration training (partly combined with resistance exercise) that was used in the two Berlin Bed Rest (BBR) studies was quite effective^20^, although there were still some BMC reductions at some of the measured sites, notably the tibia^21^. This comparison supports the mechanostat model, which states that mechanical loading of the bones is necessary and that this mechanical loading has to be high enough to cause bone deformation in a certain strain range^13^. *In-vivo* studies have shown that the strains necessary to preserve tibial bone mass according to the mechanostat model are generated during high-impact exercise such as running or jumping, whereas low-impact exercises such as cycling or even high-effort exercises such as leg press do not produce strains that are consistently above the threshold^12,22^. As expected, the pQCT measurements of the radius did not show any significant differences between groups and no main effect of time, in line with previous bed rest studies measuring the radius as an upper body control^20^. Note that the R + 14 time point for the pQCT measurements during recovery was chosen because previous bed rest studies have shown that bone loss does not stop directly after bed rest, but continues up until about two weeks after the end of the bed rest phase^20^.

The results of the maximal muscle strength tests show that the jump training was also effective in maintaining neuromuscular function of the leg extensors, while the control group showed a decrease in leg extensor strength of about 40%, in line with results from previous bed rest studies^23,24^. Even though the training consisted of highly dynamic movements, the isometric MVC tests showed no significant decrease for the training group in maximal knee extension torque and sustained knee extension torque, and only a small decrease in maximal plantar flexion torque. This transfer from dynamic training to isometric testing is not self-evident, as results from the LTBR showed: although the flywheel exercise used in that study was able to more or less maintain the training-specific performance, it was not able to prevent the decrease by about 35% in isometric leg extensor MVC^23^. The combination of flywheel and treadmill exercises used in the WISE study was more effective, but the decrease in isokinetic MVC could also not be completely prevented^24^. In BBR2, the resistive vibration training was successful in almost completely maintaining isometric knee extensor MVC, but plantar flexion MVC decreased by about 15%^25^. The finding that despite exercise, peak plantar flexion MVC decreases more after bed rest than knee extension MVC has been attributed to differences in habitual loading in daily life^26^, especially for the soleus muscle with its higher proportion of slower muscle fibres^27^. Consequently, a training program with a larger training volume is probably more suitable to maintain calf muscle strength, and a high-intensity training probably affects the gastrocnemius muscle more than the soleus muscle. Nevertheless, it is remarkable that the jump training used in the present study was able to almost maintain peak plantar flexion torque despite the low training volume. The only countermeasure that was equally effective in this domain was the combined resistance and vibration countermeasure used in BBR1 (reduction of 9% in isometric plantar flexion for the training group), which had a much higher training frequency and volume^28^.

Leg flexor strength was less affected compared to leg extensor strength. This has been reported previously^24^ and has been attributed to the higher susceptibility of “antigravity” muscles to gravitational unloading during bed rest or spaceflight^9^. This is also the reason why most exercise countermeasures have focused on the leg extensors and consequently failed to protect the leg flexors^24^. It is therefore remarkable that even though the jump training used in the present study focused on the leg extensors, it was also successful in preventing the decrease in knee flexion strength observed in the control group. One possible explanation for the efficacy of the jump countermeasure in that regard is that the leg flexors are also active during jumps: as the high forces during jumps have to be met by high joint stability to prevent injuries, co-activation of the antagonists (for example the hamstrings for the knee joint) has been proposed as one strategy to increase joint stabilization^29^, and it has been shown that jump training can increase hamstring peak torque and power^30^.

When analysing the sustained 90 s isometric knee extensions, the results replicate the results of the MVC test: a considerable decrease in the control group (about −40%) but not in the training group. Thus, the training maintained not only MVC, but also local muscle endurance in a submaximal task. As hypothesized, handgrip strength as an upper body control value was not affected by the training and also did not change after bed rest in the inactive subjects, which is in line with previous findings^31^.

The changes in leg lean tissue mass showed a similar pattern as the muscle strength tests, i.e., no decrease for the training group and a decrease for the control group. Compared to the control group’s decrease of about 40% in MVC, the loss of roughly 5% in lean leg mass is quite low though. This disproportionately higher decrease in muscle function compared to muscle mass loss has already been reported in previous unloading studies^32^. Possible explanations include alterations in neural drive and changes in muscle fibre properties, such as thin filament density^33^ and force per cross-sectional area^32^. These changes in muscle microarchitecture are not detected when using macroscopic-level imaging methods such as DXA, which consequently underestimate the loss in muscle function. Note that the training group’s seeming “loss” in lean leg tissue mass during bed rest can most likely be attributed to the well-documented increased diuresis on the first day of HDT^34^: on HDT1, 24 h urine volume increased by 1.0 ± 0.3 litre (see^35^), consistent with the seeming “loss” in lean leg mass during HDT, which also amounted to −1 ± 0.6 kg (constant for all HDT data points) and completely disappeared during recovery by reambulation-induced fluid retention.

Even though the jump training program was primarily designed to maintain leg muscle function and bone mass, it was also successful in maintaining peak oxygen uptake capacity, which declined by about 30% in the control group. This puts the efficacy of the jump training with respect to aerobic capacity on par with the efficacy of the much longer combined aerobic and resistance exercise protocol used in the WISE study: participants in the exercise group in that study had to complete 50 min of treadmill running for 2-4x per week plus about 30 min resistance training 2-3x per week, which was successful in maintaining absolute (−8%) and relative (−4%) VO2peak after 60 days of bed rest^36^. These results are similar to those of the present study, which were −8% for absolute and −5% for relative VO2peak, both not statistically significant from baseline. Unfortunately, there seem to be no publications on the effects of the resistance training used in LTBR and the combined resistance and vibration training used during BBR1 and BBR2 on aerobic capacity. However, results from shorter-duration bed rest studies suggest that resistance exercise can attenuate the decline in cardiovascular function observed in inactive controls after 30 days of bed rest by about 50%, but not prevent it^37^. Non-exercise countermeasures such as amino acid supplementation do not seem to have any positive effect on VO2peak^36^. The reason for the efficacy of the jump training program on aerobic capacity is probably its high intensity: especially the training sessions with short breaks in between series can be seen as a form of high-intensity interval training, which has been shown to increase aerobic capacity to a similar extent as higher-volume continuous endurance training with moderate intensity^38^. It has to be noted though that in contrast to BMC, BMD and MVC, aerobic capacity was not equal between groups during the baseline measurements. This result of the random subject allocation and the small sample size is a limitation of the study, but the large effect size of the interaction effect makes it unlikely that the baseline group differences had a fundamental influence on the results.

In summary, the low-volume, high-intensity jump training protocol used in the present study was very effective compared to potential countermeasures tested in previous long-term bed rest studies. It was able to maintain structure and function of three different organ systems at once (bone, muscle and cardiovascular system) with only one exercise mode (jumps), whereas other countermeasures were only effective in maintaining one or two of these three systems, even though they used a combination of exercise modes (e.g., resistance plus vibration exercise or aerobic plus resistance exercise) and had a much higher training volume or frequency. Possible reasons for the success of the jump training program include the exposition to high peak forces and rates of force development during the training (which are considered to be important for bone modelling), the high power output of the leg extensors in a whole-body movement with a large range of motion (which was probably an important factor in maintaining muscle function also in untrained tasks), and the high-intensity interval nature of the training, which might be seen as a key factor in maintaining aerobic capacity.

## Conclusion

All in all, the jump training proved to be a truly integrated effective and time-efficient countermeasure for the deteriorating effects of strict physical inactivity on tibial bone mineral content, bone mineral density, maximal leg muscle strength, local muscle endurance and leg lean mass as well as peak oxygen uptake capacity. Consequently, we suggest to verify the feasibility of jump training in at-risk populations and then incorporate it as a very time-efficient and effective type of exercise into the training programs for the elderly, astronauts on long-term space missions, and sedentary populations, especially those with a high osteoporosis risk.

## Methods

### Study design

This randomized controlled single-centre, parallel-group study with balanced randomisation was conducted at the:envihab facility of the German Aerospace Centre (DLR) in Cologne. The study was split into two campaigns with initially 12 participants each. The first campaign started in August 2015, the second campaign in January 2016. Each campaign consisted of 15 days of baseline data collection (BDC-15 through BDC-1), 60 days of strict 6° head-down tilt bed rest (HDT1 through HDT60) and 15 days of recovery (R + 0 through R + 14), see Fig. 1. During the bed rest period the subjects maintained the 6° HDT for 24 h/day. During the adaptation and recovery phases (BDC and R), physical activity was restricted to free movement within the ward. During the entire study, the subjects received a strictly controlled diet. For details, see^35^. The primary endpoint with respect to the efficacy of the training intervention was tibia bone mineral density assessed with pQCT at R + 14 compared to baseline, secondary endpoints were body lean mass according to DXA, maximal leg extensor strength during isometric knee extension and plantar flexion, and maximal aerobic capacity during a cycle step test.

### Subjects

Of the 24 healthy male subjects that were enrolled in the study, one subject discontinued the study on BDC-4 for medical reasons unrelated to the study. Subjects were randomly allocated (die roll of each pair of participants in the morning of HDT1) to either the jump training group (JUMP, 12 participants, age 30 ± 7 years, height 181 ± 7 cm and body mass 77 ± 7 kg) or the control group (CTRL, 11 participants, age 28 ± 6 years, height 181 ± 5 cm and weight 76 ± 8 kg), for details see Table 3. One participant started in the training group, but was reallocated to the control group after three training sessions due to a possible medial tibia stress syndrome. Two of the 23 subjects that completed the study (one CTRL, one JUMP) were re-ambulated after respectively 49 and 50 instead of 60 days of HDT due to medical reasons, but completed the recovery phase with all the scheduled measurements except for the spiroergometry. Before taking part in the study, all participants gave written informed consent to all the experimental procedures, which were in accordance with the relevant guidelines and regulations, and were approved by the ethics committee of the Northern Rhine Medical Association (Ärztekammer Nordrhein) in Duesseldorf, Germany, as well as the Federal Office for Radiation Protection (Bundesamt für Strahlenschutz). Inclusion criteria were as follows: male, age between 20 and 45 years, body mass index between 20–26 kg/m2, non-smoking, no medication, no competitive athlete, and no history of bone fractures. Exclusion criteria were chronic hypertension, diabetes, obesity, arthritis, hyperlipidaemia, hepatic disease (A, C), disorder of calcium or bone metabolism, or heritable blood clotting disorders. Volunteers that were medically eligible for the study subsequently underwent psychological screening, involving questionnaires and interviews. The recruitment process was concluded by a dual energy X-ray absorptiometry (DXA) screening of the bone mineral density of the femur and the lumbar vertebra column.

**Table 3:** Group characteristics at baseline.

	JUMP BDC-3	CTRL BDC-3	Group difference
Age [years]	30 ± 7	28 ± 6	p = 0.49
Height [cm]	181 ± 7	181 ± 5	p = 0.82
Body mass [kg]	78 ± 7	76 ± 8	p = 0.57
BMI [kg/m^2^]	24 ± 2	23 ± 2	p = 0.60
Body fat mass [kg]	19 ± 6	17 ± 4	p = 0.32
Body lean mass [kg]	56 ± 5	57 ± 7	p = 0.83

Group characteristics during baseline (BDC-3), separately for the training group (JUMP) and the control group (CTRL). Total body fat mass and total body lean mass were measured by DXA. There were no differences between the two groups in any of the parameters (two-tailed student’s t-tests).

### Training device

The sledge jump system (SJS, see Fig. 2) was developed by Novotec Medical GmbH (Pforzheim, Germany). It consists of a frame on wheels and a lightweight sledge that is attached to the rails. The participant is attached to the sledge via two straps around the shoulders, allowing movement in a natural manner^39,40^. The force that pulls the sledge towards the force plates is generated by low-pressure cylinders that are able to generate any force between zero and 1800 N by altering the pressure of the cylinders. During nine sessions during BDC, all subjects were familiarized with the correct jumping technique in the SJS.

### Training

The training protocol for the JUMP group during the 60 days of HDT comprised a total of 48 training sessions. On average, each session consisted of 4 × 12 countermovement jumps and 2 × 15 repetitive hops, preceded by a warm-up that consisted of 6 squats and heel raises, 3 submaximal countermovement jumps and 1 × 10 submaximal repetitive hops. All sessions were supervised and documented, and the average time spent exercising during one session was 3 minutes. Peak forces during the training amounted to 3.6 ± 0.4 kN and peak power to 3.4 ± 0.3 kW. Further details about the training can be found in^35^.

### Lean mass

DXA measurements were performed on days BDC-13, BDC-3, HDT13, HDT30, HDT45, HDT60, R + 7 and R + 14 with a Prodigy Full Pro (GE Healthcare GmbH, Solingen, Germany) using the whole body scan feature, and the manufacturer’s enCORE software (version 16.10.151) was used to generate automated reports on lean tissue mass of the legs.

### Bone

Peripheral quantitative computed tomography (pQCT) was used to measure bone mineral content (BMC) and total bone mineral density (BMD) of the left tibia with an XCT 2000 Scanner (Stratec Medizintechnik, Pforzheim, Germany) on days BDC-3 and R + 14. Cross-sectional images were obtained at 4%, 38%, 66% and 98% of the tibia length with 0% being the distal tibia end). Similarly, cross-sectional images of the radius were obtained at 60% of radius length. The pQCT images were processed with the XCT 2000 software (version 6.20B). Detection thresholds for bone mineral content were set to 280 mg/cm for the diaphysis, 181 mg/cm for the tibia epiphysis at 4%, and 120 for the tibia epiphysis at 98% (with two exceptions with a threshold of 181).

### Muscle strength

Maximal voluntary isometric torque was recorded for the following four movements using the Isomed 2000 system (D&R Ferstl GmbH, Hemau, Germany): knee extension, knee flexion, plantar flexion and dorsiflexion of the left leg. Subjects were familiarized with the equipment and procedures six days before the first measurements and then measured once directly before (BDC-1) and once directly after bed rest (R + 0). Knee extension and knee flexion torque were assessed in a sitting position with a hip angle of 90°, knee angle of 90° and ankle angle of 0°. Plantar flexion and dorsiflexion torque was assessed in a supine position with hip, knee and ankle angles of 0°. The tests were preceded by a warm-up procedure consisting 5 minutes of cycling at 75 Watt and five isokinetic submaximal contractions. Each of the four tests consisted of six repetitions of 5 s, (three repetitions separated by 1 min of rest, and after a 2 min break a second block of three repetitions).

In addition, knee extensor muscle fatigability was always assessed 5 minutes after the knee MVC using a 90-s sustained submaximal isometric contraction in the same body position as for the knee MVC tests. The target torque was set at 50% of the highest torque achieved in the preceding knee extension isometric MVC test. Subjects were instructed to quickly reach the target torque visualized on the feedback screen and maintain it for 90 s without interruptions. MVC data (analogue torque signals of the Isomed 2000) were corrected for possible offsets, followed by extraction of the maximal torque during each trial via a Matlab (Mathworks Inc, Natick, USA) script. The maximum of the six trials was used for further statistical analyses. For the 90 s knee extensor fatigability trials, the area under the torque curve was calculated. In addition to the MVC tests for the lower extremities, maximal handgrip strength of the dominant hand was recorded as a measure of upper extremity strength. For that purpose, a Jamar Plus (Patterson Medical, Warrenville, USA) hand dynamometer was used. During testing, subjects were seated with hips and knees at 90°, right arm in a vertical position (elbow and wrist extended). Three trials of five seconds each were conducted and the highest of the three results used for further analyses.

### Aerobic capacity

Peak oxygen uptake capacity (VO2peak) during cycling was measured using a cycle ergometer (Lode, Groningen, The Netherlands), once before bed rest (BDC-8) and once after bed rest (R + 1). After an initial 5 min of seated rest, subjects were instructed to start and maintain a cadence of 75 rpm while the load was increased every minute in steps of 25 W (starting from 3 min at 50 W) until volitional exhaustion despite strong verbal encouragement. Breath-by-breath oxygen uptake and carbon dioxide emission was monitored using the Innocor system (Innovision, Odense, Dänemark), heart rate was continuously monitored via 12-lead ECG (Padsy, Medset Medizintechnik, Germany). The spiroergometry data was filtered by taking the median of 5 breaths and subsequently a moving average over 30 seconds. Afterwards, the peak values for the following parameters were extracted: VO2, heart rate, respiratory exchange rate, and ergometer power. If the peak respiratory exchange rate was below 1.10, the trial was deemed not exhaustive and not considered for further analyses.

### Data availability

The data that support the findings of this study are available from the authors upon reasonable request and with permission of the European Space Agency (ESA). The data are to be made available from the Erasmus Experiment Archive (http://eea.spaceflight.esa.int/portal/) but restrictions apply to the availability of these data, which are part of the standardized ESA core data and were used with permission for the current study, and so are not publicly available. The study was registered with the German Clinical Trial Registry (DRKS, registration number DRKS00012946, 18^th^ of September 2017).

### Statistics

After verifying distribution normality with Kolmogorov-Smirnov tests, changes in response to bed rest were assessed with repeated measures analyses of variance (ANOVA), using time (BDC-3 and R + 14 for pQCT, BDC-1 and R + 0 for all strength tests, BDC-3 and R + 7 for the DXA measurements, and BDC-8 and R + 1 for spiroergometry) as repeated measure and group (JUMP, CTRL) as inter-subject factor, followed up by pre-planned post-hoc tests for the training group, alpha = 0.05. In case Mauchly’s test of sphericity produced significant results, the Greenhouse-Geisser correction was applied. Effect sizes for interaction effects were calculated via Hedge’s g (mean pre-post difference in the control group minus mean pre-post difference in the training group, divided by the pooled standard deviation). Sample size estimations were based on the results of previous bed rest studies, with an additional margin for potential dropouts (power of 0.9, alpha of 0.05, effect size of 0.4). Participants were aware of their group allocation, and no strict measures were taken to blind outcome assessors and data analysts, even though most of them were unaware of group allocation. Analyses were executed with SPSS 21.0 (SPSS, Inc., Chicago, IL). Group data are presented as means ± standard deviations (SD).
